# Screening for bone health in older people with HIV: Uptake and outcomes from a large UK outpatient HIV service

**DOI:** 10.1111/hiv.70264

**Published:** 2026-06-02

**Authors:** Rhys Nicholas, Kate Alford, Colin Fitzpatrick, Jaime Vera

**Affiliations:** ^1^ Department of Global Health and Infection Brighton & Sussex Medical School, University of Sussex Brighton UK; ^2^ The Lawson Unit, Royal Sussex County Hospital, University Hospitals Sussex NHS Foundation Trust Brighton UK

**Keywords:** bone mineral density (BMD), dual‐energy X‐ray absorptiometry (DEXA), fragility fracture, osteopenia, osteoporosis

## Abstract

**Objectives:**

Bone health is negatively affected in people with HIV, and the risks of osteoporosis and fragility fractures are increased. The objective of this study was to describe the characteristics of those accessing bone health screening and identify factors associated with osteoporosis.

**Methods:**

Retrospective data were collected from people accessing a large HIV outpatient service between September 2003 and December 2024. Inclusion criteria were age ≥ 60 years. Data were obtained from medical records, including patient demographics and clinical variables. The three most recent Dual Energy X‐ray Absorptiometry (DEXA) scans were analysed. Descriptive statistics were used to summarise patient characteristics, and logistic regression was used to identify factors associated with the development of osteoporosis.

**Results:**

Five hundred eighty‐eight people (90.1% male, 91% white, 81% Men who have sex with Men, median age 67 years) were analysed. Of these, 79.6% (*n* = 468) had at least one DEXA scan. The prevalence of osteopenia and osteoporosis was 52% (*n* = 306) and 18% (*n* = 106), respectively. Of those with osteopenia at baseline who had DEXA data available, 11.1% (*n* = 34) progressed to osteoporosis. Only 29% (*n* = 31) of individuals with osteoporosis were receiving bisphosphonate therapy. Prolonged duration on anti‐retroviral therapy, fractures and low lumbar spine T‐scores were risk factors for developing osteoporosis during follow‐up (*p* < 0.05).

**Conclusions:**

Bone health screening uptake was high in this cohort of older people with HIV, but management of established osteoporosis remained suboptimal. Previous fractures and low lumbar spine T‐scores identified those at greater risk of progression to osteoporosis.

## INTRODUCTION

People living with HIV experience a greater burden of poor bone health compared with the general population [1, 2]. Low bone mineral density (BMD) is common at HIV diagnosis and continues to worsen with ageing, co‐existing comorbidities, lifestyle factors and long‐term exposure to antiretroviral therapy (ART) [1, 3, 4]. Certain classes of ART, particularly tenofovir disoproxil fumarate (TDF) and some protease inhibitors (PI), have been associated with reduced BMD [2, 4, 5, 6]. The risk of osteoporosis is compounded by chronic immune activation, osteoblast dysregulation, inadequate bone microarchitecture and core features of metabolic syndrome, like dyslipidaemia and insulin resistance, as reported in studies investigating BMD in people with HIV [1, 7, 8, 9, 10, 11, 12]. As people living with HIV are now ageing in greater numbers, bone health has become an important factor in long‐term HIV care [1].

Guidelines for bone health assessment vary internationally [11, 13]. The British HIV Association (BHIVA) advises routine fracture risk assessment using the FRAX® tool from age 50 and recommends universal dual‐energy x‐ray absorptiometry (DEXA) scanning from age 70, regardless of additional risk factors. Earlier DEXA is recommended for individuals with an elevated FRAX® score (10‐year risk of major osteoporotic fracture ≥10%) or the presence of risk factors, such as previous fragility fracture, prolonged corticosteroid use, low BMI or previous exposure to specific ART [11]. In contrast, the European AIDS Clinical Society (EACS) focuses on a risk‐based approach starting at age 50. EACS recommends DEXA scanning for post‐menopausal women and men over 50 with additional risk factors. It is recommended that the time interval between DEXA scans, for these groups, is 3–5 years. However, there is no age threshold for universal screening [13]. These differing approaches reflect the ongoing uncertainty in the literature about the optimal timing and strategy for bone health assessment in people with HIV [14, 15, 16]. Furthermore, this uncertainty was reflected in research showing that reliance on the FRAX® score to determine DEXA eligibility meant that most with established osteoporosis were missed because of the weak correlation between FRAX® score and DEXA result [17].

Identifying and managing low BMD is important because people living with HIV experience a higher rate of fragility fractures compared to HIV‐negative adults [1, 9, 12, 13]. The definition of a fragility fracture is *“a fracture sustained from a force equivalent to a fall from a standing height or less”* [18]. EACS recommends that all patients with low BMD (T‐score ≤ −2.5) are at risk of fragility fracture and should be considered for bisphosphonate therapy [13].

The aims of this study were to identify the characteristics and the frequency of bone health screening among people with HIV at a local healthcare service (Brighton, UK) and to identify factors associated with progression to osteoporosis.

## MATERIALS AND METHODS

### Study design

Retrospective data were collected from a cohort of people living with HIV, aged ≥60 years old, accessing a local HIV outpatient service in Brighton, UK, from September 2003 to December 2024. Brighton has the oldest cohort of people with HIV in Europe, with 70% (*n* = 2500) over 50 years old. Electronic patient medical records were used to collect the data, and an audit documented information about patient demographics, past medical historyize and previous engagement with BMD screening. DEXA scans were performed at the Brighton and Sussex Clinical Trials Unit, or Sussex Medical Chambers, using the ‘*Hologic Discovery C'* model.

Medical variables included comorbidities, frailty status, measured using the FRAIL scale [19] and previous fractures of the vertebrae, upper limbs or lower limbs. Comorbidity data were obtained from routinely recorded diagnoses in electronic medical records. HIV‐specific variables included length of time living with HIV, type and length of time taking ART and viral load.

### Bone health parameters

Variables pertaining to DEXA included the number of scans, T‐scores, FRAX® scores and bisphosphonate treatment. T‐scores were analysed from the lumbar spine (L1‐L4), hip and femoral neck. Results of the scans were determined by following the EACS and WHO definitions of BMD disorders [13, 18]. A normal result was defined as a T‐score ≥ −1.0 standard deviation (SD). Osteopenia was defined as a T‐score less than −1.0 SD but greater than −2.5 SD [18]. Osteoporosis was defined as a T‐score ≤ −2.5 SD [13, 18]. FRAX® scores were calculated using the gold‐standard algorithm from the University of Sheffield [20].

### Statistical analysis

Categorical variables with different values were coded appropriately, and continuous variables were given a measurement scale. The prevalence of BMD disorders was calculated by considering the T‐scores at all three anatomical sites. Time between HIV diagnosis and baseline DEXA, and time between subsequent DEXA scans, were calculated in years. DEXA‐1 corresponds to the baseline DEXA scan, DEXA‐2 to the second available scan, and DEXA‐3 to the third available scan, with observed intervals reported between scans.

Logistic regression was used across all DEXA scans to identify factors associated with having osteoporosis (T‐score ≤ −2.5 SD). We used duration living with HIV in initial models, and selected duration on ART exposure for the final logistic regression models. Kaplan–Meier curves were used to illustrate survival time (time to osteoporosis) when adjusting for different independent variables, such as age. Two‐sided *p‐*values ≤0.05 were statistically significant. All statistical analyses were performed using IBM SPSS Version 29.

This was a retrospective service‐based evaluation using anonymised routinely collected data, and according to the Health Research Authority in the UK, formal research ethics committee review and individual participant consent were not required.

## RESULTS

### Characteristics of people with HIV undertaking bone health screening

588 people with HIV, aged 60 years and over, were included in the analyses. 90.1% (*n* = 530) were male. 91% were white (*n* = 535). 81% (*n* = 476) were men who have sex with men. The median age was 67 years (range 60–98). The median length of time spent living with HIV was 23.3 years (range 2–44), and 98% (*n* = 576) had an undetectable viral load. The median CD4 count was 646 cells/mm^3^ (range 62–1956).

In terms of risk factors for BMD disorders, with respect to ART exposure, 20.2% (*n* = 119) of the cohort were receiving a TDF‐containing regimen at the time of data collection, including 10.7% (*n* = 63) on a regimen containing TDF and a PI. Overall, 73.8% (*n* = 434) had been switched away from TDF‐containing ART. 92.5% (*n* = 98) of those with osteoporosis had been switched away from TDF, and no participant with osteoporosis remained on TDF. 48.3% (*n* = 284) were taking an Integrase Strand Transfer Inhibitor (INSTI) class of antiretroviral, and 2.2% (*n* = 13) were on this medication in combination with a PI.

12.8% (*n* = 75) were frail, and the prevalence of diabetes and falls was 16.7% (*n* = 98) and 11.1% (*n* = 65), respectively. The prevalence of all fractures, irrespective of mechanism and site, was 29.9% (*n* = 176) (Table 1). The characteristics of individuals who had received a DEXA scan are presented in Table 2. When comparing the characteristics of those who received a DEXA scan to those who did not, the demographics are similar, although there was a slightly higher prevalence of frailty and previous fractures in the DEXA scan group (Tables 1 and 2).

**TABLE 1:** Participant demographics for the whole cohort.

Variable	Total (*N* = 588)
Demographic data	
Age (years)*	67 (60–98)
Cis‐male (%)	530 (90.1)
White (%)	535 (91)
Black British/African (%)	40 (6.8)
Mixed Ethnic Background (%)	9 (1.5)
MSM (%)	476 (81)
HIV clinical data	
Time since HIV diagnosis (years)*	23.3 (2–44)
CD4 count (cells/mm^3^)	646 (62–1956)
Duration of exposure to ART (years)*	20.4 (2–37)
VL <40 at first assessment (%)	576 (98)
Current exposure to TDF (%)	119 (20.2)
ART switch away from TDF (%)	434 (73.8)
Health data	
Frailty status	
Robust (%)	310 (52.7)
Pre‐frail (%)	109 (18.5)
Frail (%)	75 (12.8)
Previous fractures (%)	176 (29.9)
No previous fractures (%)	412 (70.1)
Comorbidities	
CVD (%)	380 (64.6)
Diabetes (%)	98 (16.7)
Falls (%)	65 (11.1)
Renal Disease (%)	160 (27.2)
None (%)	15 (2.6)

*Note*: All values are expressed as n, unless stated otherwise.

Abbreviations: CVD, cardiovascular disease; MSM, men who have sex with men; TDF, Tenofovir Disoproxil Fumarate; VL, viral load; ART, antiretroviral therapy.

* Median (range).

**TABLE 2:** Baseline characteristics of those individuals with available DEXA data.

Variable	Total (*N* = 468)
Demographic data	
Age (years)*	68 (60–98)
Cis‐male (%)	416 (88.9)
White (%)	426 (91)
Black British/African (%)	33 (7.1)
Mixed Ethnic Background (%)	5 (1.1)
MSM (%)	380 (81.2)
HIV clinical data	
Time since HIV diagnosis (years)*	23.7 (2–44)
CD4 count (cells/mm^3^)	691 (93–1956)
Duration of ART (years)*	20.8 (2–37)
VL <40 at first assessment (%)	463 (98.9)
Current exposure to TDF (%)	89 (19)
ART switch away from TDF (%)	354 (75.6)
Health data	
Frailty status	
Robust (%)	241 (51.5)
Pre‐frail (%)	90 (19.2)
Frail (%)	66 (14.1)
Previous fractures (%)	153 (32.7)
No previous fractures (%)	315 (67.3)
Comorbidities	
CVD (%)	303 (64.7)
Diabetes (%)	74 (15.8)
Falls (%)	59 (12.6)
Renal Disease (%)	129 (27.6)
None (%)	10 (2.1)

*Note*: All values are expressed as n, unless stated otherwise.

Abbreviations: ART, antiretroviral therapy; CVD, cardiovascular disease; MSM, men who have sex with men; TDF, Tenofovir Disoproxil Fumarate; VL, viral load.

* Median (range).

### Screening uptake

Of the 588 participants in the cohort, 79.6% (*n* = 468) underwent at least one DEXA scan. Among those who had an initial scan, 66.9% (*n* = 313) went on to have a second DEXA scan, and 53.7% (*n* = 168) had a third scan. When comparing the cohort which received a follow‐up DEXA to those who did not, the populations were similar with respect to demographics (Table 3). Importantly, the comparison table suggests that those with osteoporosis were more likely to have a follow‐up scan, and those with normal BMD were less likely to be followed up.

**TABLE 3:** Comparison of characteristics between individuals with and without follow‐up DEXA scans.

Variable	Follow‐up DEXA (*N* = 313)	No Follow‐up DEXA (*N* = 155)
Demographic data		
Age (years)*	68 (60–98)	66 (61–92)
Cis‐male (%)	277 (88.5)	139 (89.7)
White (%)	285 (91.1)	141 (91)
Black British/African (%)	23 (7.3)	10 (6.5)
Mixed Ethnic Background (%)	4 (1.3)	1 (0.6)
MSM (%)	260 (83.1)	120 (77.4)
HIV clinical data		
Time since HIV diagnosis (years)*	24.6 (3–44)	22.1 (2–41)
CD4 count (cells/mm^3^)	690 (93–1956)	691 (157–1700)
Duration of ART (years)*	22.1 (3–37)	18.8 (2–36)
VL <40 at first assessment (%)	311 (99.4)	152 (98.1)
Current exposure to TDF (%)	55 (17.6)	34 (21.9)
ART switch away from TDF (%)	244 (78)	110 (71)
Health data		
Frailty status		
Robust (%)	150 (47.9)	91 (58.7)
Pre‐frail (%)	61 (19.5)	29 (18.7)
Frail (%)	53 (16.9)	13 (8.4)
Previous fractures (%)	98 (31.3)	55 (35.5)
No previous fractures (%)	215 (68.7)	100 (64.5)
Comorbidities		
CVD (%)	209 (66.8)	90 (58.1)
Diabetes (%)	52 (16.6)	22 (14.2)
Falls (%)	41 (13.1)	18 (11.6)
Renal Disease (%)	87 (27.8)	42 (27.1)
None (%)	5 (1.6)	5 (3.2)
Bone Mineral Density		
Normal	72 (23)	56 (36.1)
Osteopenia	173 (55.3)	80 (51.6)
Osteoporosis	53 (16.9)	19 (12.3)

*Note*: All values are expressed as n, unless stated otherwise.

Abbreviations: ART, antiretroviral therapy; CVD, cardiovascular disease; MSM, men who have sex with men; TDF, Tenofovir Disoproxil Fumarate; VL, viral load.

* Median (range).

Following the baseline DEXA scan, 13% (*n* = 61) had a FRAX® score ≥ 10%. Of these, 62.3% (*n* = 38) had follow‐up imaging. Among those who had a second DEXA scan, 16.6% (*n* = 52) had a FRAX® score ≥ 10%, of whom 36.5% (*n* = 19) proceeded to a further scan. The proportion of participants with FRAX® scores ≥10% increased across successive assessments, from DEXA‐1: 13% (*n* = 61) to follow‐up; 16.6% (*n* = 52) at DEXA‐2 and 18.5% (*n* = 31) at DEXA‐3.

After the first DEXA scan, the overall median time to follow‐up was 3.0 years (range 0.4–15.6). The overall median time to receive a third scan was 3.9 years (range 0.4–13.2). For individuals with a normal baseline DEXA result (T‐score ≥ −1), the mean time to repeat scan was 3.6 years (± 2.8 years), which was consistent with guideline recommendations of 3 to 5 years [13]. In contrast, mean follow‐up intervals were not shorter for those with abnormal baseline BMD, measuring 3.9 years (± 2.9) in those with osteopenia and 3.7 years (± 2.3) in those with osteoporosis.

### Changes in bone mineral density and fracture risk

Among participants who received a baseline DEXA scan (*n* = 468), 54.1% (*n* = 253) had osteopenia, and 15.4% (*n* = 72) had osteoporosis at any anatomical site. Of those with osteopenia at baseline who had follow‐up DEXA scan results available, 11.1% (*n* = 34) progressed to osteoporosis. In the cohort with osteoporosis (*n* = 106), only 29.2% (*n* = 31) were receiving bisphosphonate therapy.

The femoral neck consistently had the lowest median T‐scores (baseline: −1.2; follow‐up: −1.4 and − 1.5), while the lumbar spine had the greatest range of T‐scores (baseline: 8.5; follow‐up: 8.7 and 9.0). Older age and previous fractures were associated with lower T‐scores during follow‐up (*p* < 0.05).

Previous fractures were also consistently associated with higher FRAX® scores (*p* < 0.05), and the median FRAX® score increased modestly over time (baseline 5.8%; follow‐up: 6.6% and 6.7%).

However, the FRAX® score showed poor sensitivity. Among the 72 people who had a DEXA‐confirmed diagnosis of osteoporosis and available FRAX® data at baseline, only 37.7% (*n* = 23) recorded a score ≥10%.

### Factors associated with progression to osteoporosis

Two logistic regression models identified baseline lumbar spine and hip T‐scores, and duration of ART as the strongest predictors of developing osteoporosis during follow‐up (*p* < 0.05). Frailty status was identified as a potential contributor to bone health in this cohort (*p* < 0.05) (Table 4). Participants' sex was not a significant factor in the model. There was no difference between men and women in terms of progression to osteoporosis (*p* = 0.20).

**TABLE 4:** Logistic regression models investigating factors associated with osteoporosis.

Model 1 (*n* = 227)					
Variable	B	SE	*p*‐value	Exp (B)	95% CI
Duration of ART (years)	0.077	0.066	0.239	1.080	0.950–1.229
Total number of comorbidities	0.174	0.212	0.411	1.190	0.786–1.804
Lumbar spine T‐score (DEXA‐1)	−5.370	1.562	<0.001	0.005	0.000–0.099
Hip T‐score (DEXA‐1)	−4.494	1.947	0.021	0.011	0.000–0.507
Femoral neck T‐score (DEXA‐1)	0.345	1.360	0.800	1.412	0.098–20.290

Model 2 (*n* = 137)					
Variable	B	SE	*p*‐value	Exp (B)	95% CI
Frailty status	4.319	1.790	0.016	75.085	2.248–2508.087
Duration of ART (years)	0.222	0.096	0.021	1.249	1.034–1.508
Total number of comorbidities	0.117	0.237	0.622	1.124	0.706–1.787
Lumbar spine T‐score (DEXA‐2)	−3.189	0.967	<0.001	0.041	0.006–0.275
Hip T‐score (DEXA‐2)	−0.833	1.082	0.441	0.435	0.052–3.622
Femoral neck T‐score (DEXA‐2)	−3.782	1.698	0.026	0.023	0.001–0.635

*Note*: *p*‐value, significance *p* ≤ 0.05.

Abbreviations: 95% CI, 95% confidence interval; ART, antiretroviral therapy; B, regression coefficient; DEXA 1, baseline scan; DEXA 2, a follow‐up scan; Exp(B), odds ratio; SE, standard error.

Higher T‐scores of the lumbar spine and hip, at baseline, were protective factors against having osteoporosis during follow‐up. Lumbar spine T‐scores had the strongest effect. In contrast, longer cumulative exposure to ART and frailty were associated with greater odds of osteoporosis. The odds ratio for frailty was large, but the wide confidence interval suggests that this estimate should be interpreted cautiously due to a small sample size (Table 4). Therefore, the effect of frailty on BMD in this cohort should be considered an exploratory finding as opposed to a key predictor of bone health.

**FIGURE 1:**
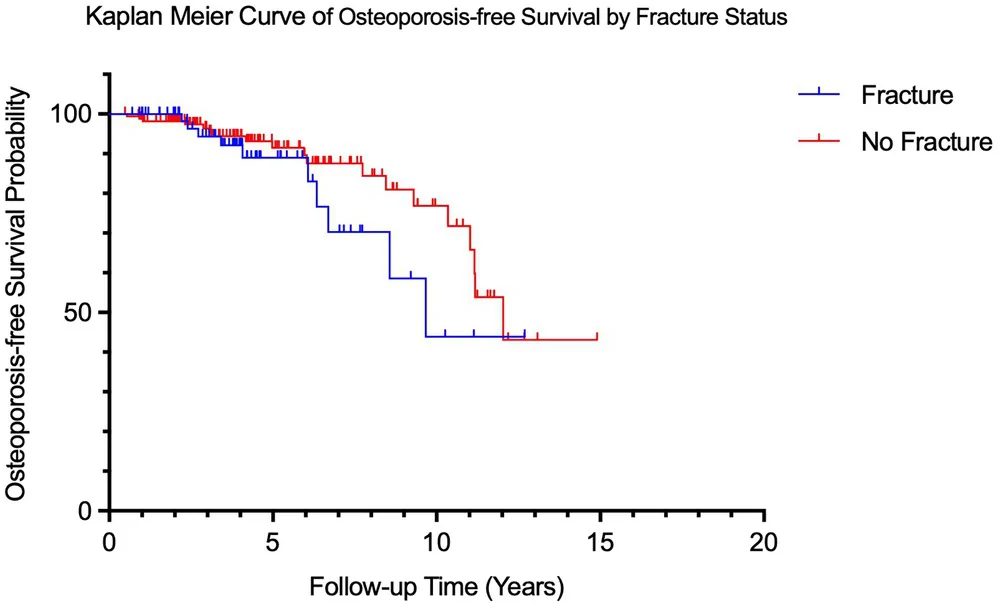
Kaplan–Meier curve showing osteoporosis‐free survival according to fracture status. The curves show the probability of remaining free from osteoporosis during follow‐up.

Together, these models demonstrated high specificity (96–97.5%) and moderate sensitivity (59%–72%). Kaplan–Meier analysis supported these findings; individuals with a previous fracture progressed to osteoporosis during follow‐up more rapidly than those without (*p* < 0.05) (Figure 1, Table 5).

**TABLE 5:** Number at Risk (Number Censored During Time Interval).

No Fracture	171 (34)	134 (52)	78 (30)	45 (16)	27 (10)	15 (6)	5 (3)	1 (1)
Fracture	72 (13)	59 (23)	32 (16)	15 (6)	6 (1)	3 (2)	1 (1)	–
Overall	243 (47)	193 (75)	110 (46)	60 (22)	33 (11)	18 (8)	6 (4)	1 (1)
	**0**	**2**	**4**	**6**	**8**	**10**	**12**	**14**

*Note*: Time to Follow‐up DEXA Scan (Years).

## DISCUSSION

This study investigated bone health in older adults living with HIV and the factors associated with low BMD. We found that decreased BMD was common; over half of the cohort had osteopenia (52%) and nearly one in five had osteoporosis (18%). A meaningful minority progressed to osteoporosis during follow‐up (*n* = 34). Previous fractures and low lumbar spine T‐scores at baseline were the strongest predictors of deterioration, highlighting the importance of early identification and targeted monitoring.

These findings align with previous work showing high rates of bone loss among people with HIV. The reported prevalence of osteopenia was between 63% and 67%, and osteoporosis between 12% and 15% [2, 17]. A meta‐analysis had also shown significantly lower lumbar spine BMD and higher vertebral fracture prevalence in people with HIV compared to HIV‐negative controls [1]. Together with our results, this emphasises the value of lumbar spine BMD as a sensitive marker for early bone disease and a priority site for ongoing assessment.

A strength of this cohort was the high uptake of DEXA scanning; 79.6% received at least one scan. Despite this, screening gaps remained, as more than one in five individuals had never undergone a scan. Adherence to guideline‐recommended follow‐up was mixed. The average time to follow‐up for people with normal BMD was appropriate. However, follow‐up time for those with abnormal BMD was not shorter, despite their higher risk profile. Furthermore, only 62.3% of those with an elevated FRAX® score (≥10%) went on to receive follow‐up imaging, indicating missed opportunities for fracture‐risk management and incomplete adherence to guidelines [11, 13]. Improving engagement with bone health assessments remains an important service goal.

Medication review also contributed to reducing risk. Most individuals who had been previously exposed to TDF (73.8%) were switched to alternative ART regimens, and no participant with osteoporosis remained on TDF at the time of data collection. This is consistent with awareness of the potential impact of TDF on bone health and with contemporary prescribing practice [4, 6].

This study has several limitations. The cohort was predominantly comprised of white men, reflecting local service demographics but limiting generalisability to women and people of Black, Asian and mixed ethnic backgrounds, who may have different risk profiles. Another important limitation is the inclusion criteria of ≥60 years, rather than the ≥50 years used in guidelines. The age threshold of 60 years old was chosen to focus the study on the older segment of our clinic population, in whom bone health concerns are most prevalent. Exclusion of those aged 50–59 years may limit the clinical applicability of the results and reduce the comparability with other studies [11, 13].

Missing and incomplete data posed additional challenges. While information about important clinical variables was complete, frailty status, a potentially important contributor to BMD decline, was missing in 16% of participants, increasing the risk of bias [21, 22]. No analysis on ART subtypes was carried out in our study because this detailed information was not collected. The effect of ART on bone health was examined more broadly, and as such, this is a limitation of our study and an area for development. In addition, fracture data were sometimes incomplete, as electronic records did not consistently document the mechanism of injury. As a result, some fractures may have been misclassified as fragility fractures and incorrectly entered into the FRAX® calculator, potentially leading to overestimated risk scores.

An important finding was the low proportion of people with osteoporosis who received bisphosphonate treatment (29.8%). This is clinically relevant because current guidelines recommend considering anti‐resorptive therapy in individuals with osteoporosis, fragility fracture, or elevated fracture risk [11, 13, 23]. Our findings suggest that identification of low BMD through DEXA did not consistently translate into treatment. The reasons for this could not be determined from this retrospective dataset and warrant further investigation to identify barriers to access and uptake. This represents an important area for service improvement, as bone health surveillance is of limited value if it is not linked to appropriate management. Bone disease progression was assessed only descriptively across available follow‐up scans in the retrospective cohort, and more advanced longitudinal modelling was beyond the scope of the present study. Although FRAX® formed part of routine fracture risk assessments, we did not evaluate concordance between FRAX® and DEXA findings in great detail. The analysis we did perform showed that FRAX® was not a sensitive diagnostic tool for osteoporosis in our cohort. This is an important area of future research, as FRAX® may under‐identify some individuals with clinically important bone disease in people with HIV. Also, vertebral fractures were not systematically assessed, and this may have led to underestimation of fracture burden and affected the interpretation of FRAX® and lumbar spine findings. Finally, follow‐up DEXA uptake declined across successive scans, and we did not formally compare those with and without imaging. These findings should therefore be interpreted with caution. Repeat scanning in clinical practice is influenced by multiple factors, including patient attendance, competing clinical priorities, clinician decision making, service capacity and costs.

Despite these limitations, prolonged duration of ART exposure was associated with BMD decline, while frailty appears to be a possible contributing factor. These findings suggest that individuals who have lived longest with HIV, who are often older and more comorbid, should be prioritised for closer monitoring. Importantly, recovery of the lumbar spine T‐score appeared unlikely in this population, and a low baseline lumbar spine T‐score was strongly predictive of subsequent osteoporosis. This is supported by other research, which found that people with HIV had lower BMD of the lumbar spine and a greater prevalence of vertebral fractures compared to HIV‐negative controls [1]. Clinicians should therefore treat low lumbar spine measurements as an early warning sign and manage these individuals proactively.

Future research should explore the barriers that people with HIV face when accessing bone‐health screening, as improving DEXA uptake could improve early detection and management. Further investigation into the vulnerability of the lumbar spine in this population is also warranted to understand the mechanisms driving bone loss at this site.

In conclusion, uptake of bone health screening in this cohort of older adults with HIV was high, but important gaps remain in follow‐up and treatment. Individuals with previous fractures and low lumbar spine T scores appeared to be at greater risk of progression to osteoporosis. These findings support a more targeted approach to bone health management. Individuals with established osteoporosis or fragility fractures should receive appropriate treatment and risk factor modification, while those at increased risk of progression may benefit from closer monitoring and preventive intervention.

## AUTHORS CONTRIBUTIONS

*Conceptualisation*: Rhys Nicholas, Kate Alford, Jaime Vera. *Formal analysis*: Rhys Nicholas. *Investigation*: Rhys Nicholas, Colin Fitzpatrick. *Resources*: The Lawson Unit, Brighton Sexual Health and Contraception Service, Royal Sussex County Hospital, Brighton and Sussex Clinical Trials Unit, Sussex Medical Chambers. *Supervision*: Kate Alford and Jaime Vera. *Writing—original draft*: Rhys Nicholas. *Writing—review & editing*: Rhys Nicholas, Kate Alford, Professor Jaime Vera.

## CONFLICT OF INTEREST STATEMENT

Dr. Kate Alford has been an investigator on studies and received funding from ViiV Healthcare and Gilead Sciences Ltd. Professor Jaime Vera has received honoraria and research grants, been a consultant or investigator in trials sponsored by Merck, Janssen Cilag, Piramal and Gilead Sciences. He has received sponsorship to attend scientific conferences from Janssen Cilag, Gilead Sciences and AbbVie.

## Data Availability

The data that support the findings of this study are available from the corresponding author upon reasonable request.
